# The Importance of a Gatekeeper Residue on the Aggregation of Transthyretin: IMPLICATIONS FOR TRANSTHYRETIN-RELATED AMYLOIDOSES*

**DOI:** 10.1074/jbc.M114.563981

**Published:** 2014-08-01

**Authors:** Ricardo Sant'Anna, Carolina Braga, Nathalia Varejão, Karinne M. Pimenta, Ricardo Graña-Montes, Aline Alves, Juliana Cortines, Yraima Cordeiro, Salvador Ventura, Debora Foguel

**Affiliations:** From the ‡ Instituto de Bioquímica Médica Leopoldo de Meis, Programa de Biologia Estrutural,; the ‖ Faculdade de Farmácia, and; the ¶ Instituto de Microbiologia Professor Paulo de Goés, Universidade Federal do Rio de Janeiro, Rio de Janeiro CEP 21941-590, Brazil and; the § Institut de Biotecnologia i Biomedicina, Universitat Autònoma de Barcelona, 08193 Bellaterra, Spain

**Keywords:** Amyloid, Heparin, Peptides, Protein Aggregation, Protein Misfolding, Aggregation Propensity, Gatekeeper Residue, Rational Mutation, Transthyretin

## Abstract

Protein aggregation into β-sheet-enriched amyloid fibrils is associated with an increasing number of human disorders. The adoption of such amyloid conformations seems to constitute a generic property of polypeptide chains. Therefore, during evolution, proteins have adopted negative design strategies to diminish their intrinsic propensity to aggregate, including enrichment of gatekeeper charged residues at the flanks of hydrophobic aggregation-prone segments. Wild type transthyretin (TTR) is responsible for senile systemic amyloidosis, and more than 100 mutations in the TTR gene are involved in familial amyloid polyneuropathy. The TTR 26–57 segment bears many of these aggressive amyloidogenic mutations as well as the binding site for heparin. We demonstrate here that Lys-35 acts as a gatekeeper residue in TTR, strongly decreasing its amyloidogenic potential. This protective effect is sequence-specific because Lys-48 does not affect TTR aggregation. Lys-35 is part of the TTR basic heparin-binding motif. This glycosaminoglycan blocks the protective effect of Lys-35, probably by neutralization of its side chain positive charge. A K35L mutation emulates this effect and results in the rapid self-assembly of the TTR 26–57 region into amyloid fibrils. This mutation does not affect the tetrameric protein stability, but it strongly increases its aggregation propensity. Overall, we illustrate how TTR is yet another amyloidogenic protein exploiting negative design to prevent its massive aggregation, and we show how blockage of conserved protective features by endogenous factors or mutations might result in increased disease susceptibility.

## Teaser

**Background:** Proteins have adopted negative design to diminish aggregation.

**Results:** The replacement of Lys-35 by Leu increases the amyloidogenicity of the 26–57 segment of TTR as well as the entire protein.

**Conclusion:** Lys-35 is as a gatekeeper residue in TTR, and its protective effect is suppressed by heparin.

**Significance:** The elucidation of the principles that govern protein aggregation is helpful for the design of strategies against amyloid diseases.

## Introduction

Amyloid deposits have been associated with pathological states such as Alzheimer and Parkinson diseases and familial amyloid polyneuropathy (FAP),^3^ among others (1–3). In these diseases, a specific soluble protein or peptide undergoes structural changes, forming aggregation-prone, β-sheet enriched species, which constitute the building blocks for amyloid fibril formation. Toxicity in these diseases arises either by the loss of function of the aggregated protein(s) or by a gain of toxic function associated with the presence of fibrils themselves or prefibrillar assemblies, which, as suggested by several studies, are cytotoxic (4–6).

The aggregation of proteins into amyloid fibrils has been proposed to be an intrinsic property of polypeptide chains, and indeed an increasing number of proteins unrelated to disease are being shown to form amyloids *in vitro* (7, 8). This reflects the fact that structural and sequential determinants of protein folding overlap significantly with those promoting intermolecular aggregation (9–12). Therefore, during evolution, proteins have adopted negative design strategies to counter the unavoidable aggregation propensity of certain sequence stretches by incorporating β-sheet breakers at structurally critical positions (13, 14), avoiding the presence of β-strands on the edge of protein structures (15) or placing gatekeeper residues at the flanks of aggregation-prone segments (16–18). Gatekeeper residues counteract aggregation by the repulsive effect of their charges (Arg, Lys, Asp, and Glu), by the entropic penalty on aggregation due to their large and flexible side chains (Arg and Lys), and by the incompatibility with the β-sheet structure of the aggregates (Pro). The impact of gatekeeper residues on the aggregation tendency of proteins can be large; a single substitution of a hydrophobic residue by a Lys suffices to fully convert a highly aggregation-prone protein into a soluble variant (19).

Transthyretin (TTR) is a homo-tetrameric protein of 56 kDa produced in the liver and in cerebrospinal fluid. TTR is associated with at least two amyloidoses: senile systemic amyloidosis (SSA) and FAP. SSA is caused by massive deposition of aggregates of wild type TTR (WT-TTR), mainly in the heart, whereas FAP is caused by deposition at peripheral nerves and tissues of aggregates that are composed of more than 100 different autosomal TTR variants (20, 21). Aggregation of TTR is triggered by low pH (22, 23) and initiated by the dissociation of tetramers into partially unfolded monomers, which are inherently aggregation-prone (23–25). At present, liver transplantation is the only treatment for FAP. Tafamidis, a drug that kinetically stabilizes the TTR tetramer, has been approved in Europe to treat V30M FAP patients. However, little is known about the efficacy of tafamidis in non-V30M FAP as well as in other TTR-related amyloidoses, involving cardiac and neurological manifestations (1, 26). Thus, additional studies are necessary to unravel the precise mechanism of TTR aggregation and to develop effective therapies for these pathologies.

**FIGURE 1.**
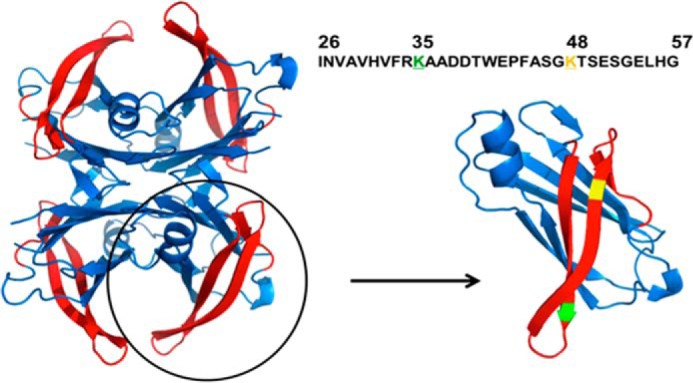
**Structural and sequential features of the TTR 26–57 region.** On the *left*, the tetrameric TTR structure is displayed with the β-strands B, C, and D, comprising residues 26–57, *highlighted* in *red* (Protein Data Bank entry 1F41). On the *right* is shown the 26–57 peptide sequence used in the present study and the monomer of TTR with lysines 35 and 48 *colored* in *green* and *yellow*, respectively.

Here we combined *in silico* and *in vitro* analyses to explore whether TTR exploits gatekeeper residues to modulate its propensity to form aggregates, focusing on the predictably amyloidogenic 26–57 stretch comprising strands B, C, and D (Fig. 1, *red strands*). Several studies have shown that strands C and D participate in the interchain contacts that lead to fibril formation in TTR (27–29). Many FAP mutations map to strands C and D, including V30M and L55P, the most widespread and aggressive TTR variants, respectively (30, 31). Despite these features, we show here that, surprisingly, this region displays a low propensity to aggregate *in vitro*. We identify Lys-35 as a gatekeeper residue responsible for this behavior because the mutation K35L within a synthetic TTR 26–57 peptide or in the complete tetrameric protein yields a large increase in amyloidogenicity.

Heparin promotes fibrillation of WT-TTR and co-localizes with the protein in the heart tissue of SSA patients (32). This proamyloidogenic effect depends on its binding selectively to a basic motif among residues 24–35 of strand B (32). Accordingly, we show that heparin bypasses the gatekeeping effect of Lys-35. Thus, our results demonstrate that TTR exploits negative design strategies to diminish its intrinsic propensity to aggregate and that perturbation of these protective features can trigger its amyloidogenic potential.

## EXPERIMENTAL PROCEDURES

### 

#### 

##### AGGRESCAN, TANGO, and AMYLPRED2 Analysis

The intrinsic aggregation and amyloid formation propensities were evaluated for the wild type TTR sequence and the K35L and K48L variants, either in the context of the complete protein sequence or considering the isolated TTR 26–57-derived peptides. These analyses were performed with the AGGRESCAN, TANGO, and AMYLPRED2 algorithms, employing default settings.

##### Peptide Synthesis

The three peptides studied here were purchased from Genemed Synthesis with a purity of 99.9%. The peptide compositions were confirmed by MALDI-TOF/TOF mass spectrometry.

##### Protein Expression and Purification

WT-TTR and the K35L-TTR mutant were expressed and purified as described previously (31). Protein concentration was determined by absorbance spectroscopy using an extinction coefficient of 7.76 × 10^4^ m^−1^ cm^−1^ at 280 nm.

##### Peptide Aggregation

Desired amounts of lyophilized peptides were weighted and dissolved on a known volume of Milli-Q water for preparation of fresh 1 mm stock solutions just before the aggregation experiments. Peptide concentration was also confirmed by spectroscopy, measuring the absorbance of the stock solutions at 280 nm and using an extinction coefficient of 5690 m^−1^ cm^−1^. The concentration used for aggregation experiments was 100 μm in all assays. Buffers were named as buffer pH 5.0 (100 mm MES, 100 mm KCl, pH 5.0) and buffer pH 7.5 (50 mm Tris, 100 mm KCl, pH 7.5). Samples were incubated without agitation at 25, 37, and 50 °C. The aggregation kinetics were followed by measuring the suspension turbidity (at 330 nm) along the kinetics or at the indicated reaction time points using a UVI-Vis Mini-1240 Shimadzu spectrophotometer (Shimadzu).

WT peptide aggregation was also studied in the following buffers: 10 mm HCl, 5 mm SDS (buffer 1); 10% acetonitrile (buffer 2); 50% TFE (buffer 3); 50% DMSO (buffer 4); 10 mm HCl, 20% TFE (buffer 5); 200 mm sodium acetate, pH 5.0 (buffer 6); and 5 mm Triton X-100 (buffer 7).

##### Thioflavin T (Th-T) Binding Assay

Th-T binding was used to probe amyloid formation on the samples. Peptide samples were diluted to 5 μm in a Th-T solution in 5 mm sodium phosphate, pH 8, resulting in a final Th-T concentration of 20 μm. Excitation was set to 450 nm, whereas emission was collected from 400 to 600 nm. Five individual scans were averaged for each measurement using a K2 spectrofluorometer (ISS Inc.). The intensity of the spectra at 482 nm was used as an indicator of the extent of amyloid material. Specifically, in Figs. 5*A* and 8*B*, the suspensions containing the aggregated material (12 or 18 h of aggregation, respectively) were centrifuged at 11,000 rpm for 15 min, the pellets were resuspended in buffer, and equal amounts of aggregates were mixed with Th-T to measure binding.

##### Full-length TTR Aggregation Assay

3.5 μm WT-TTR and K35L-TTR native proteins were incubated under quiescent conditions in 200 mm sodium acetate, 100 mm KCl, pH 4.4, at 37 °C. After 72 h, samples were vortexed, and their turbidity (330 nm) was monitored on a UVI-Vis Mini-1240 Shimadzu spectrophotometer (Shimadzu).

##### Circular Dichroism (CD) Measurements

Samples of the three peptides (WT, K35L, and K48L) were prepared immediately before measurements at a concentration of 100 μm by dissolving the stock solution on buffer pH 5.0 or 7.5. CD spectra were recorded at the indicated times, scanning from 260 to 190 nm on a Jasco 810 spectropolarimeter thermostated at 25 °C. Each spectrum results from the accumulation of 10 scans. When aggregation of the WT peptide was performed in 10 mm HCl, 5 mm SDS (buffer 1), CD measurements were also collected at the same conditions.

##### Transmission Electron Microscopy (TEM)

Aggregated samples were diluted to 10 μm in Milli-Q water. 5 μl of suspension were absorbed onto 200-mesh carbon-coated copper grids for 5 min and then blotted to remove excess material. Negative staining was performed by adding 5 μl of 2% (w/v) uranyl acetate. Samples were dried on air for 3 min. The grids were imaged with a Jeol 1200 electron microscope (Jeol Ltd.) operating at a 60 kV acceleration voltage.

##### Proteinase K (PK) and Trypsin-limited Proteolysis for Determination of Amyloid Fibril Core

Samples of K35L peptide fibrils grown in buffer pH 5.0 at 25 °C for 3 h were centrifuged for 15 min at 11,000 rpm. The pellet was carefully resuspended in the same volume of PBS, pH 7.0. Digestion was initiated by adding PK or trypsin at a final concentration of 2 μg/ml. PK proteolysis was stopped after 5, 30, or 60 min by the addition of PMSF to attain a final concentration of 5 mm. 50 μl of the digestion mixture were immediately diluted into 200 μl of PBS, pH 7, containing 9 m urea and incubated for 2 h to allow denaturation of the remaining aggregates. The trypsin reaction was quenched by the addition of 1% TFA. TFA at this concentration is effective in both inactivating this enzyme and completely resolubilizing the remaining aggregates (33). Digestion products were separated by a Superdex peptide (GE Healthcare), and elution was followed by recording absorbance at 214 nm. The resistant core was considered to be a peak for which no change in intensity was detected along the digestion reaction. The peaks were collected, and the solvent was removed by centrifugal evaporation. The resulting pellets were resuspended in 0.1% TFA and further concentrated using ZipTip pipette tips (Millipore). Samples were then mixed with the same volume of α-cyano matrix and spotted immediately for mass spectrometry analysis. Spectra were collected using an Ultraflex Speed MALDI-TOF/TOF system (Bruker Daltonics). Data were extracted and exported using the program mMass.

##### Size Exclusion for Oligomeric State Characterization

The oligomeric state of K35L peptide at 0, 5, 10, 15, and 30 min after initiation of the aggregation reaction in buffer pH 5.0 at 25 °C was evaluated using SEC by injecting 50 μl of each sample onto a Superdex 75 column (GE Healthcare) coupled to a HPLC system (Shimadzu). Sample elution was followed by absorbance at 280 nm.

##### Fibril Characterization by FTIR

Aggregated samples were centrifuged for 15 min at 11,000 rpm. The resulting pellets were resuspended in D_2_O and lyophilized overnight. Samples were analyzed by attenuated total reflection-FTIR spectroscopy using a Magna 550 FTIR spectrophotometer (Nicolet) equipped with a mercury cadmium telluride detector. Infrared (IR) spectra were obtained by co-adding 256 interferograms collected with 2 cm^−1^ resolution and apodized with a Happ-Genzel function. Determination of peak positions and curve fitting were performed with the OMNIC (Nicolet) and GRAMS (Galactic) software, respectively. Integral intensities of the secondary structure elements were calculated by analysis of the amide I vibrational mode of the infrared spectrum. Fourier self-deconvolution of the IR spectra was performed with a resolution enhancement factor of 1.8 and a bandwidth of 15 cm^−1^.

##### Seeding Experiments

Seeding of the K35L peptide aggregation kinetics were assayed by adding known amounts of nuclei solution (K35L peptide suspension previously aggregated for 20 min at pH 5.0 in buffer pH 5.0 at 25 °C) or 2% (v/v) sonicated mature fibrils to freshly prepared samples of peptide at 100 μm. The percentage of nuclei indicated on the experiments was calculated as v/v. The aggregation kinetics of the K35L peptide under different seeding conditions were followed by recording turbidity at 330 nm over time.

##### Aggregation in the Presence of Heparin

The effect of heparin on WT peptide aggregation was evaluated by incubating samples of peptide in buffer pH 5.0 or pH 7.5 in the presence of low molecular weight heparin at 10, 20, or 30 μm final concentration. The peptide concentration was fixed at 100 μm. The aggregation kinetics were followed by recording the turbidity of the samples at 330 nm. A sample of the peptide incubated in the absence of heparin was used as a negative control. In the case of the full-length TTR (WT and K35L, 3.5 μm), 35 μm heparin was employed.

##### High Hydrostatic Pressure-induced Denaturation

Pressure-induced denaturation of WT- and K35L-TTR (full-length proteins) was followed by recording tryptophan emission spectra between 300 and 400 nm after excitation set to 280 nm. Pressure was applied with increments of 200 bars, using a K2 spectrofluorometer (ISS) coupled to a high pressure cell described previously (34). For spectrum recording, the sample was allowed to equilibrate for 15 min after each pressurization step. The degree of denaturation of the protein was evaluated using the center of spectral of mass of the tryptophan emission at each pressure.

## RESULTS

### 

#### 

##### The Aggregation Propensity of the TTR 26–57 Segment Is Intrinsically Low but Can Be Artificially Induced by Additives

Initially, we incubated the peptide encompassing residues 26–57 of wild type human TTR (WT peptide) at 100 μm in a large variety of experimental conditions, including extreme pH (2.0–12.0), high temperatures (up to 60 °C), and high salt concentrations (up to 500 mm KCl). However, we could not detect any significant peptide aggregation as monitored by light scattering, Th-T binding, or electron microscopy (EM) in any of these conditions (data not shown).

**FIGURE 2.**
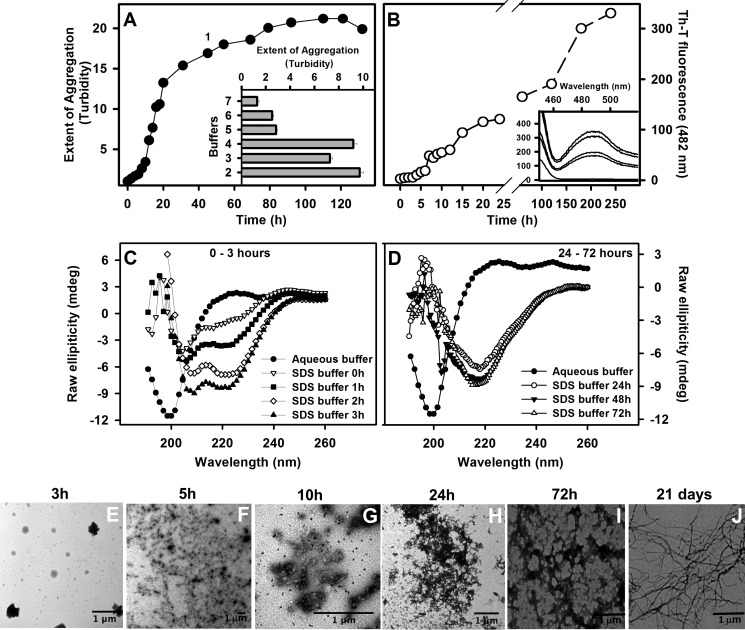
**Characterization of the WT peptide aggregation.** *A*, the WT peptide was incubated at 100 μm at room temperature with different buffers and aggregation monitored by turbidity at 330 nm (*inset*). Turbidity was normalized by the initial values obtained for each sample. The constitution of each buffer is as follows: 10 mm HCl, 5 mm SDS (buffer 1); 10% acetonitrile (buffer 2); 50% TFE (buffer 3); 50% DMSO (buffer 4); 10 mm HCl, 20% TFE (buffer 5), 200 mm sodium acetate, pH 5.0 (buffer 6); and 5 mm Triton X-100 (buffer 7). *B* shows the Th-T binding assay performed with the sample incubated on HCl-SDS buffer at different times. Th-T fluorescence was obtained by exciting the samples at 440 nm and collecting the emission from 400 to 600 nm. The intensity at 482 nm was used to probe amyloid formation. The *inset* shows examples of Th-T fluorescence emission spectra. *C* and *D*, secondary structural changes during the aggregation kinetics in HCl-SDS as monitored by CD, showing the transient population of α-helical conformers at initial aggregation times (*C*) and the conversion toward a β-sheet-rich conformation at 24 h (*D*). TEM was performed with samples aggregated in SDS for 3 h (*E*), 5 h (*F*), 10 h (*G*), 24 h (*H*), 72 h (*I*), and 21 days (*J*). All *bars* in the *images* represent 1 μm. *Error bars*, S.E.

To force aggregation of this peptide, we used seven buffers containing different additives (acetonitrile, TFE, SDS, Triton X-100, and DMSO) and monitored the extent of aggregation by turbidity after 120 h (Fig. 2*A*, *inset*; see the figure legend for buffer constitution). All of them were effective in inducing peptide aggregation to some extent. However, the most pronounced aggregation effects were observed when the peptide was incubated in the presence of 50% DMSO (buffer 4), 50% TFE (buffer 3), 10% acetonitrile (buffer 2), and 10 mm HCl and 5 mm SDS (buffer 1). The latter condition was chosen for a better characterization of the aggregation process of WT peptide. As seen in Fig. 2*A* (*filled circles*), in this condition, aggregation of WT peptide presented a lag phase of ∼8 h in duration followed by an exponential growth phase that levelled off at ∼80 h, when turbidity increased 20-fold. It is interesting to note that a non-ionic surfactant, such as Triton X-100 (buffer 7), did not induce a substantial induction on the aggregation of the WT peptide, which suggests that charged surfactants are necessary for this effect.

In order to probe the amyloid character of the detected aggregates of WT peptide in HCl-SDS (buffer 1), aggregation kinetics measuring Th-T were performed (Fig. 2*B*). For this, peptide aliquots were withdrawn during the aggregation kinetics and mixed with Th-T, and the fluorescence spectra were recorded (Fig. 2*B*, *inset*). It is possible to see an increase in fluorescence emission at 482 nm over time, suggesting formation of amyloid aggregates. And, again, a nucleation phase in the first ∼5 h was noticed, followed by an exponential growth phase.

We monitored simultaneously the change in the secondary structure content of the peptide using far-UV CD (Fig. 2, *C* and *D*). Although the peptide comprises the B, C, and D β-strands in the context of the intact protein, in the absence of additives, it exhibited a main peak at 200 nm, characteristic of an unstructured conformation (*filled circles* in *both panels*). The presence of SDS induced a progressive shift of the spectrum toward that of an α-helix, with two negative signals at 208 and 222 nm. This conformational transition was observable immediately after dilution in SDS, indicating a fast induction of α-helical contacts. At later times, 1, 2, and 3 h after dilution, the intensity of these peaks increased (Fig. 2*C*).

Incubation in this buffer for 24 h resulted in a dramatic shift in the spectrum, which now presented a single intense negative peak at 216 nm, indicating a transition toward formation of β-sheet-enriched conformations (Fig. 2*D*). The negative intensity of this peak continued to increase after 48 and 72 h.

We used TEM to confirm the morphological properties of the aggregates formed upon incubation for 3, 5, 10, 24, and 72 h and 21 days in SDS (Fig. 2, *E–J*, respectively). After 3 h of aggregation (nucleation phase, where α-helical species are present), we could not see any aggregated material in suspension (Fig. 2*E*). At 5 and 10 h (end of the lag phase), amorphous aggregates were formed, and they responded for the majority of the species present (Fig. 2, *F* and *G*). At longer incubation times, fibrillar structures were observed, but they became well structured only after prolonged incubation (Fig. 2, *H-J*); unfortunately, the high viscosity of the sample after prolonged incubation prevented spectroscopic analysis of the peptide conformation at these stages.

##### Lys-35 Is Predicted to Act as Gatekeeper Residue in WT Peptide

**FIGURE 3.**
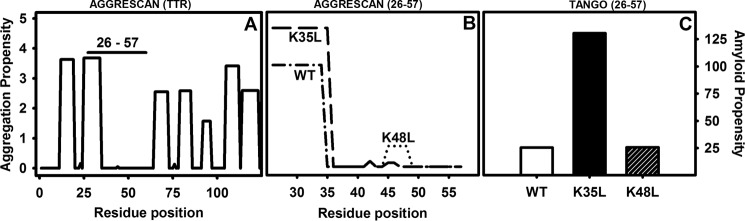
***In silico* predictions of WT-TTR and 26–57 peptide amyloid propensity.** The presence of hot spots of aggregation was predicted using AGGRESCAN for the entire TTR sequence (*A*) or for the peptides WT, K35L, and K48L (*B*). The *horizontal bar* in *A* indicates the localization of the peptides studied here in the context of TTR full-length sequence. The global aggregation propensity of WT, K35L, and K48L peptides was predicted using TANGO (*C*). The default parameters of the algorithms were used for all of the predictions.

The rationalization of protein aggregation reactions has made it possible to develop computational algorithms able to predict the aggregation propensity of polypeptide chains with good accuracy. AMYLPRED2 is an algorithm that exploits 11 of these individual programs to provide consensus predictions (35). To our surprise, seven of the predictors coincided to indicate that the region between Ile-26 and Arg-34 has a significant aggregation propensity when considered in the context of the full-length protein (Fig. 3*A*) as well as in the 26–57 peptide (Fig. 3*B*). Therefore, we speculated that there must be additional sequential/conformational contributions that counterbalance the intrinsic aggregation tendency of this sequence, allowing the WT peptide to remain soluble in aqueous solution.

Protein evolution exploits gatekeeper residues to confront the unavoidable aggregation propensity of certain protein sequences. Thus, the presence of two consecutive charged lysine residues adjacent to an aggregation-prone region has been shown to prevent amyloid formation in Src homology 3 domains, whereas mutation of one of these residues to leucine triggers their aggregation (7, 36, 37). Interestingly, the residue in position 35 in WT peptide is a lysine, which together with arginine at position 34 places two positively charged residues flanking the predicted aggregation-prone region (Fig. 1). To test whether Lys-35 might act as a gatekeeper residue as lysine does in Src homology 3 domains, we virtually mutated this residue to Leu and assessed the theoretical impact of the mutation on the peptide aggregation propensity using the above-mentioned algorithms. They consistently predicted an extension of the aggregation-prone region to include Leu-35 (Fig. 3*B*). Both the AGGRESCAN and TANGO (38, 39) programs predicted a significant increase in the aggregation propensity of the peptide upon K35L mutation (Fig. 3, *B* and *C*). To ensure that this effect was sequence-specific and not only attributable to a change in the net charge of the peptide, we exploited the presence of an additional Lys residue at position 48 in the peptide and simulated its mutation to Leu. According to both AGGRESCAN and TANGO, the K48L mutation would have a negligible effect on the aggregation of the peptide (Fig. 3, *B* and *C*).

##### Conformational Properties of TTR 26–57 Peptides

**FIGURE 4.**
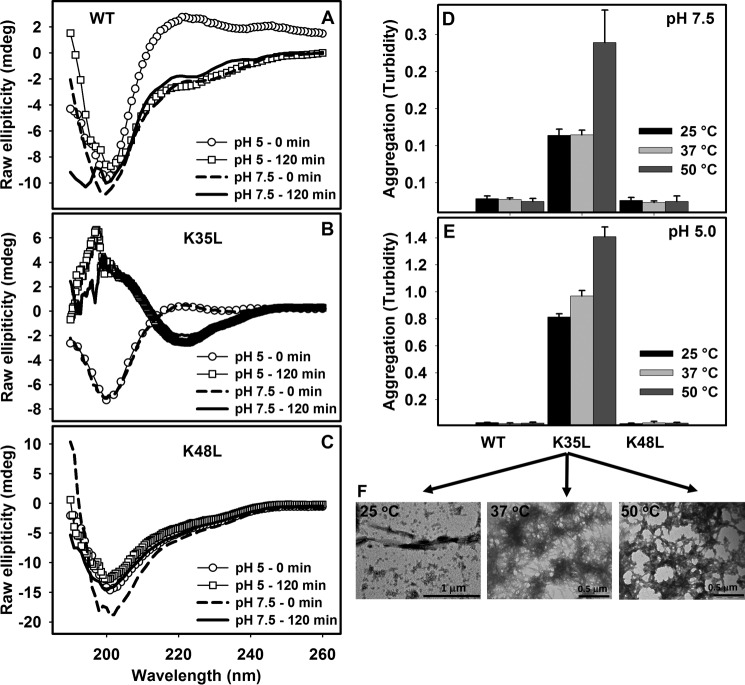
**Secondary structure determination and aggregation properties of WT, K35L, and K48L 26–57 peptides.** CD spectra of the WT (*A*), K35L (*B*), or K48L (*C*) peptides immediately after dilution in buffers pH 5.0 (*circles*) or pH 7.5 (*dashed lines*). The peptides were allowed to aggregate at 25 °C for 120 min at pH 5 (*squares*) and pH 7.5 (*continuous line*), and their CD spectra were recorded. Shown are end points of aggregation reactions of the three peptides at 100 μm for 12 h at pH 7.5 (*D*) or pH 5.0 (*E*) at 25 °C (*black bar*), 37 °C (*light gray bar*), and 50 °C (*dark gray bar*) as measured by absorbance at 330 nm. *F*, TEM images of the aggregates of K35L formed at pH 5.0 at 25, 37, and 50 °C (from *left* to *right*, respectively). *Bars*, 1 or 0.5 μm, as indicated. *Error bars*, S.E.

To validate the above described predictions, we synthesized two additional peptides encompassing TTR residues 26–57 bearing either the K35L or the K48L substitution and compared their properties experimentally with those of the wild type sequence. To evaluate the secondary structure content of the three sequences, we conducted far-UV CD experiments at pH 5.0 and 7.5. The peptide samples were prepared at 100 μm in the corresponding buffers at 25 °C. The initial spectra were recorded immediately after dilution of the peptides. In these conditions, all of the peptides exhibited spectra consistent with essentially unordered conformations, displaying an intense negative peak at around 200 nm at both pH values (see spectra in *empty circles* or *dashed lines* in Fig. 4, *A–C*). However, only K35L exhibited dramatic spectroscopic differences upon incubation for 120 min at 25 °C (Fig. 4*B*). In excellent agreement with the predictions, the peptide K35L displayed a spectrum with a negative peak at 216 nm at both pH values, indicating a transition toward a β-sheet-rich structure (Fig. 4*B*), whereas the WT (Fig. 4*A*) and the K48L (Fig. 4*C*) peptides remained in a random coil-like conformation.

##### Amyloid Properties of TTR 26–57 Peptides

To compare the aggregation properties of the WT, K35L, and K48L peptides, we incubated them at 100 μm for 12 h at 25, 37, and 50 °C at pH 7.5 (Fig. 4*D*) or pH 5.0 (Fig. 4*E*). Aggregation, as measured by turbidity at 330 nm, varied drastically among the three sequences. The K35L peptide solution underwent a large increase in turbidity upon incubation at both pH values, indicating the formation of macromolecular aggregates at different temperatures. The largest turbidity increase was observed at pH 5.0 at all temperatures and mainly at 50 °C. In contrast, the WT and K48L peptide solutions remained crystal clear and did not exhibit any increase in turbidity at any temperature at these two pH values.

Aggregates formed by the K35L peptide at pH 5.0 at different temperatures were analyzed by TEM. The images indicate that the reaction temperature affected the macromolecular organization of the aggregates, which displayed different morphologies. Well organized fibrillar structures characteristic of amyloids were observed at 25 °C, although some amorphous aggregates were also present. A progressive coalescence of the aggregates occurred as temperature increased (Fig. 4*F*).

**FIGURE 5.**
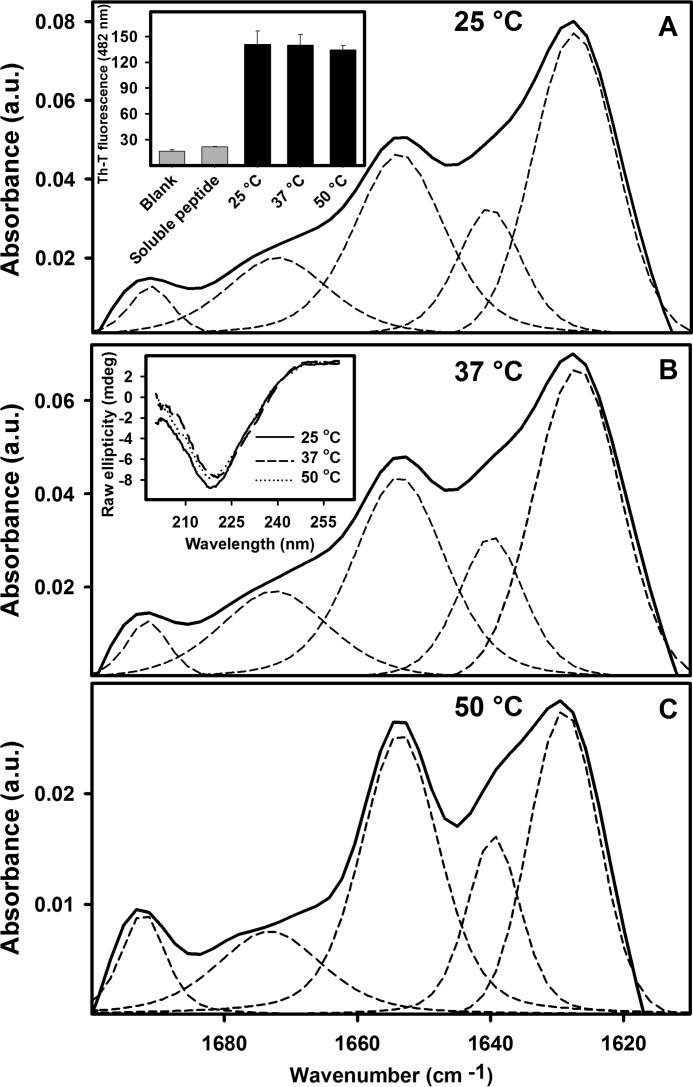
**Comparing the secondary structure of the aggregates composed of K35L peptide as measured by FTIR.** 100 μm K35L peptide was allowed to aggregate for 12 h at pH 5.0 at 25 °C (*A*), 37 °C (*B*), or 50 °C (*C*). *Dashed lines*, deconvolution of each spectrum (see also Table 1). The *insets* in *A* and *B* show, respectively, Th-T binding assays and CD spectra (*continuous*, *dashed*, and *dotted lines* are for 25, 37, and 50 °C, respectively) of the aggregates formed at these three temperatures. *a.u.*, absorbance units. Error bars, S.E.

FTIR spectroscopy in the amide I region of the spectrum has proved to be a powerful tool for the investigation of structural differences in aggregated proteins (40, 41). Experiments were performed with the insoluble fraction of the samples composed of the K35L peptide, isolated by centrifugation, and subsequently washed with D_2_O. Fig. 5, *A–C*, shows the absorbance FTIR spectra of the aggregated material and the deconvolution of the spectra into its main contributions. Despite certain conformational differences, all aggregates shared the two peaks characteristic of a β-sheet intermolecular structure at 1620–1630 cm^−1^ and 1690–1695 cm^−1^ (Table 1).

**TABLE 1:** **Secondary structure content of K35L fibrils** Data were extracted from the deconvoluted FTIR spectra shown in Fig. 5.

Structural assignment	25 °C		37 °C		50 °C	
	Wave number	Area	Wave number	Area	Wave number	Area
	*cm*^−*1*^	%	*cm*^−*1*^	%	*cm*^−*1*^	%
β-Sheets	1692, 1617	9	1691	1	1695	2
β-Sheets	1625	15	1630	9	1628	21
Turns	1677	14	1675	5	1663, 1678	29
Disordered	1640	19	1643	38	1642	18
α-Helix/random	1655	44	1659	47	1653	31

We used Th-T binding and far-UV CD to further analyze the conformational properties of these assemblies. Despite their different macroscopic aspects, the aggregates that formed when K35L peptide was incubated at different temperatures bound similar amounts of Th-T (Fig. 5*A*, *inset*) and displayed a characteristic β-sheet CD spectrum with a single negative peak at 220 nm of similar intensity (Fig. 5*B*, *inset*).

It is important to mention that, as we did before with the WT peptide (Fig. 2, *A* and *B*), we tested several different additives to foster aggregation of the K48L peptide without success (not shown). Neither HCl-SDS was effective in this case (data not shown).

##### Temperature Dependence of the Aggregation Kinetics of the K35L Peptide

**FIGURE 6.**
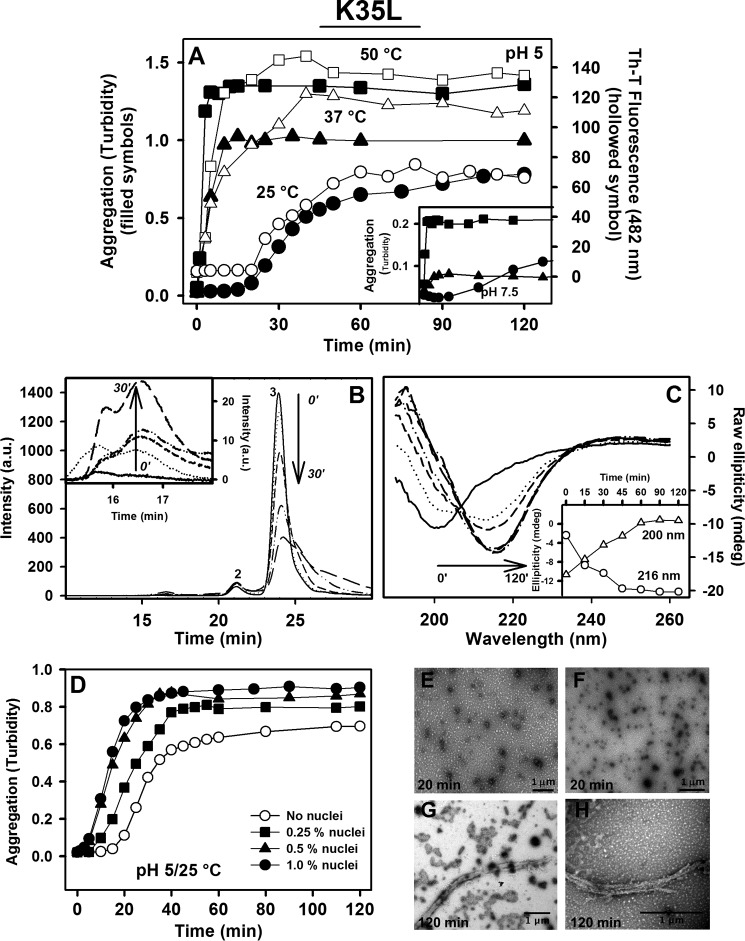
**Characterization of the K35L peptide aggregation kinetics.** A 100 μm concentration of the K35L peptide was incubated at pH 5.0 (*A*) or pH 7.5 (*inset*) at 25 °C (*circles*), 37 °C (*triangles*), or 50 °C (*squares*), and absorbance at 330 nm (*filled symbols*) or Th-T binding (*hollow symbols*) was followed over time. *B*, following aggregation of K35L peptide at pH 5.0, 25 °C by SEC. Samples were withdrawn at 0, 5, 15, and 30 min of aggregation and injected onto a Superdex 75 column, and the absorbance at 280 nm was monitored. *Peak 3* (24.7 min) corresponds to the elution of the peptide in the soluble, monomeric state. The species eluting in *peak 1* (16.8 min; details in *inset*) increased with incubation time. *C*, CD was used to follow the changes in secondary structure occurring during fibril formation at pH 5.0 and 25 °C. The *inset* shows the changes in signals at 200 and 216, which indicate the conversion from random coil to a β-sheet-rich structure. *D*, preformed nuclei accelerate aggregation of K35L peptide at pH 5.0 and 25 °C in a concentration-dependent manner (*hollow circles*, unseeded reaction; *squares*, 0.25% nuclei; *triangles*, 0.5% nuclei; *filled circles*, 1.0% nuclei (v/v)). *E* and *F*, TEM images of the species present at 20 min of the aggregation reaction (nuclei); *G* and *H*, fibrils formed at the end of the aggregation reaction (120 min). *Bars*, 1 μm.

We further evaluated the time course of the K35L peptide aggregation reaction under the conditions described above by monitoring the changes in turbidity and Th-T binding with time (Fig. 6*A*, *filled* and *hollow symbols*, respectively). The previously observed temperature dependence of aggregation at pH 5.0 was also evident at the kinetic level. The maximal extent of aggregation was achieved after ∼60, 20–30, and 20 min at 25, 37, and 50 °C, respectively. At 25 °C, the aggregation reaction followed a sigmoid curve, and the same type of kinetics was observed when the peptide was incubated at 25 °C and pH 7.5 (Fig. 6*A*, *inset*). In contrast, independently of the pH, the aggregation of the peptide at both 37 and 50 °C corresponded to an exponential reaction, without any detectable lag phase.

##### Formation of Oligomers in the Aggregation Reaction of the K35L Peptide

To detect the presence of typical amyloid oligomers and their characteristics during amyloidogenesis of the K35L peptide, we induced its aggregation at pH 5.0 and 25 °C and monitored the process during the lag phase by SEC (Fig. 6*B*) and far-UV CD (Fig. 6*C*). Aliquots of the reaction were injected at 5, 10, 15, and 30 min onto a Superdex 75 column, and the absorbance of the sample was monitored at 280 nm (Fig. 6*B*). As a control, a sample of the soluble WT peptide at the same concentration was injected. The main peak, with a retention time of 24.7 min, corresponds to the soluble peptide, and as expected, its intensity decreased with incubation time. Two other peaks were observed on the chromatograms, at 21 and 16.8 min. The peak at 21 min did not evolve with time and was already present in the control. In contrast, the peak at 16.8 min was absent in the control, increased with time, and corresponded to a peptide assembly with a molecular mass of ∼40 kDa (Fig. 6*B*, *inset*). Although there was a notable increase in the peak related to the aggregates, it is possible that part of these species is retained inside the column. Analysis of the same reaction by CD (Fig. 6*C*) showed that the transition from a disordered to a β-sheet-rich structure was fast and already complete at 45 min (*inset*) with no additional enhancement of the β-sheet content at later times.

It is well known that the addition of nuclei or seeds accelerates amyloid formation (42, 43). To test if this was the case for the K35L peptide oligomeric assemblies, we incubated the peptide at pH 5.0 and 25 °C for 20 min, added different amounts of this incubated peptide to freshly prepared peptide samples, and monitored the aggregation reaction by following the changes in turbidity (Fig. 6*D*). The presence of 0.5 or 1.0% (v/v) preformed oligomeric species had a dramatic effect on the aggregation reaction, and the lag phase was virtually abolished. The high potency of the oligomeric species to induce aggregation was evident from the fact that the addition of 0.25% preincubated peptide sufficed to reduce the lag phase to one-fourth of its original duration. We also observed an increase in the total extent of aggregation after 120 min, which was proportional to the amount of oligomers used in the assay.

We used TEM to monitor the morphology of the species promoting this seeding effect. As seen in Fig. 6, *E* and *F*, they correspond to small, spherical, and, in a few cases, “wormlike” aggregates, a morphology consistent with that of the oligomeric species of other amyloidogenic proteins described in the literature (44–46). At the end of the aggregation process of a seeded aggregation reaction (120 min), the sample presented fibrillar aggregates (Fig. 6, *G* and *H*).

Importantly, when we tried to seed the aggregation reaction with different amounts of seeds generated by sonication of K35L peptide mature fibrils formed at pH 5.0 and 25 °C, we could not see any accelerating effect (data not shown), which indicates that at least for this particular peptide, it is the oligomers, and not the fractured mature fibrillar structure, that provide the template for the initial aggregation steps.

##### Leu-35 Is Embedded in the Core of K35L Peptide Fibrils

With the demonstrated importance of substituting Leu for Lys for the aggregational properties of the region 26–57, we decided to analyze whether Leu-35 was part of the K35L fibril core. After incubation of K35L peptide for 2 h at 25 °C, the fibrils were treated with PK or trypsin (2 μg/ml) for 5, 30, and 60 min, and the protected regions were purified by SEC and identified by mass spectrometry (Table 2). The experimental details of the fibrils' digestion and purification by SEC are described under “Experimental Procedures.” We detected a segment spanning the region 34–44 that was not accessible to PK digestion even after 60 min of reaction. It has to be emphasized that the soluble peptide treated with PK under the same conditions was completely digested after 5 min (not shown). When the same experiments were repeated using trypsin as a protease, we observed a larger but overlapping fragment encompassing residues 35–57, in which Lys-48 (a potential cleavage site), was protected from digestion (Table 2). Thus, in both cases, Leu-35 mapped to the protected region of the peptide in the fibrillar state.

**TABLE 2:** **Leu-35 is embedded in the core of K35L peptide fibrils** K35L fibrils formed at pH 5.0 and 25 °C were submitted to limited proteolysis using PK or trypsin. After digestion, the fragments were purified by SEC and sequenced by mass spectrometry. The sequence of the entire 26–57 peptide before digestions is INVAVHVFRLAADDTWEPFASG**K**TSESGELHG, where leucine 35 is underlined and the boldface lysine is the theoretical trypsin cleavage site that is protected in the fibrillar state.

Protease	Protected core	Theoretical mass	Experimental mass	Error
		*Da*	*Da*	*Da*
PK	RLAADDTWEPF	1320.4	1320.6	0.2
Trypsin	LAADDTWEPFASG**K**TSESGELHG	2405.5	2405.1	0.4

##### K35L-TTR Is as Stable as WT-TTR but Has Enhanced Aggregation Propensity

**FIGURE 7.**
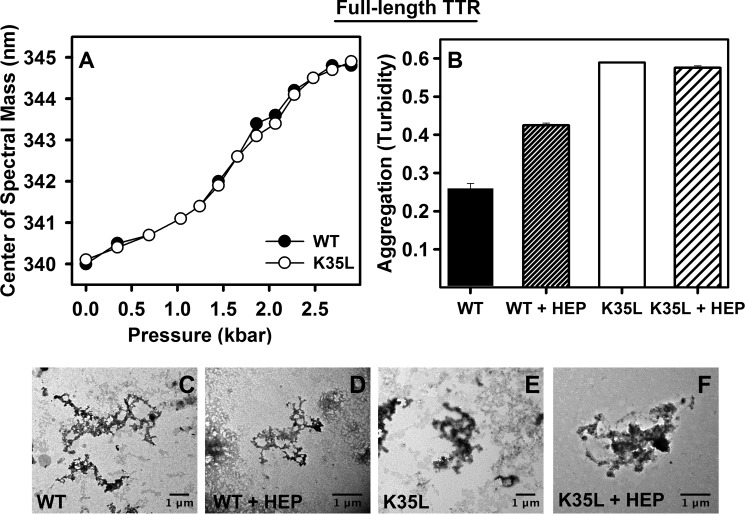
**Comparing stability and aggregation propensity between WT- and K35L-TTR.** *A*, 1 μm WT-(*filled circle*) or K35L-TTR (*hollow circle*) were subjected to high hydrostatic pressure, and tryptophan fluorescence emission was collected to evaluate the extent of dissociation-denaturation. The experiments were performed at 25 °C and pH 7.5. *B*, acid-induced aggregation (3.5 μm, pH 4.4, 37 °C for 72 h) of WT- and K35L-TTR in the absence (*solid bars*) or presence of 35 μm heparin (*hatched bars*) as followed by absorbance at 330 nm. *C–F*, TEM images of the aggregates composed of the WT- or K35L-TTR grown in the absence (*C* and *E*, respectively) or in the presence of heparin (*D* and *F*, respectively). *Bars*, 1 μm. *Error bars*, S.E.

We addressed the effect of the K35L mutation on the stability of tetrameric TTR. To this end, the wild type and mutant recombinant proteins were expressed and purified, and their thermodynamic properties were studied by using high hydrostatic pressure as a perturbing tool to induce dissociation-denaturation of the protein (47, 48). Proteins (1 μm) were incubated at pH 7.5 and 25 °C, and tryptophan fluorescence emission was used as a probe of tetramer integrity (49, 50). As shown in Fig. 7*A*, both dissociation-unfolding curves overlapped, suggesting that the introduction of a Leu at position 35 did not alter the stability of the TTR tetramers. Computational analysis using the FoldX force field (53) also predicted identical stabilities for the two variants (data not shown).

Next, we analyzed the impact of the K35L mutation on the aggregation of tetrameric TTR by incubating the WT and mutant proteins under conditions known to induce TTR amyloidogenesis (3.5 μm at pH 4.4 for 72 h at 37 °C) (22, 51). As can be observed in Fig. 7*B* (*solid bars*), the aggregation extent of K35L-TTR was more than twice that exhibited by WT-TTR under the same conditions, thus recapitulating the peptide results and reinforcing the role of Lys-35 as a gatekeeper residue even in the context of the full-length protein.

##### Heparin Bypasses the Gatekeeping Effect of Lys-35 at Mild Acidic pH

**FIGURE 8.**
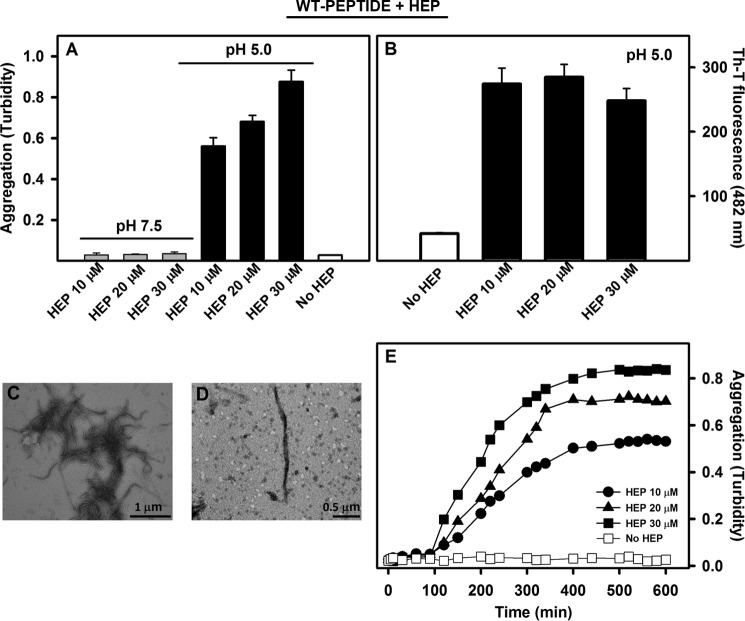
**Heparin-induced aggregation of WT peptide is pH-dependent.** *A* and *B*, 100 μm WT peptide was allowed to aggregate at 25 °C for 18 h at pH 5.0 (*black bars*) or pH 7.5 (*gray bars*) in the absence or presence of heparin (10, 20, or 30 μm as mentioned), and the absorbance at 330 nm (*A*) or Th-T binding (*B*) was evaluated. *C* and *D*, TEM images of the fibrils grown in the presence of 30 μm heparin. *Bars*, 1 and 0.5 μm, as indicated. *E*, WT peptide aggregation kinetics at pH 5.0 and 25 °C induced by increasing heparin concentrations, as followed by absorbance at 330 nm (*filled circles*, 10 μm heparin; *triangles*, 20 μm heparin; *squares*, 30 μm heparin). Note that WT peptide does not aggregate in the absence of heparin (*hollow squares*). *Error bars*, S.E.

Heparin and heparan sulfate are acidic glycosaminoglycans. These molecules are formed by unbranched repeating units of disaccharides and can be sulfated to different extents. Heparan sulfate and especially heparin facilitate TTR fibrillogenesis *in vitro* and *in vivo*, although the exact mechanism underlying this effect is still under debate (32, 52, 53). Therefore, we investigated whether heparin had any influence on the aggregation properties of this TTR region. We incubated the WT peptide at 100 μm at pH 5.0 and 7.5 in the absence or presence of 10, 20, and 30 μm low molecular weight heparin and monitored aggregation by turbidity. Fig. 8*A* shows the end points of aggregation after 18 h at 25 °C. The samples incubated at pH 7.5 did not display any significant aggregation, whereas a large increase in turbidity was observed at pH 5.0 in a heparin concentration-dependent manner. As presented above, in the absence of heparin, the WT peptide did not exhibit any aggregation propensity at pH 5.0 (Figs. 2*A* (*buffer 6*) and 8*A*). We used Th-T and TEM to probe the amyloidogenic character of the aggregates formed in the presence of heparin. As shown in Fig. 8*B*, significant Th-T binding could be observed only when the WT peptide was incubated in the presence of heparin. Aliquots of the Th-T-positive aggregates formed in the presence of 30 μm heparin were imaged by TEM (Fig. 8, *C* and *D*). The aggregates displayed the well organized fibrillar structure characteristic of amyloids. Next, we evaluated the time course of the WT peptide aggregation in the presence of different concentrations of heparin (Fig. 8*E*). At all heparin concentrations, the aggregation reaction could be fitted to a sigmoid curve characterized by three kinetic stages: 1) lag phase, 2) exponential growth phase, and 3) plateau phase. These phases, characteristic of most amyloid processes, indicate that the presence of heparin induces the aggregation of the WT peptide through a nucleation-polymerization mechanism (54, 55).

Next, we investigated whether heparin would increase the aggregation of wild type and K35L full-length TTR. In accordance with previous studies (32), heparin indeed enhanced the aggregation of wild type TTR at pH 4.4 (Fig. 7*B*, *hatched bars*). However, this effect of heparin was not observed with K35L-TTR, suggesting the participation Lys-35 in heparin-TTR interaction and aggregation enhancement. TEM shows that the aggregates grown in the absence or presence of heparin have the same fibrillar appearance (Fig. 7, *C–F*).

## DISCUSSION

Although WT-TTR is an amyloidogenic protein causing SSA, here we showed that Lys-35 protects the region 26–57, which has a highly amyloidogenic stretch, and even the entire protein against a massive aggregation, which would render the SSA an early onset disease. The importance of the 26–57 region to TTR aggregation was demonstrated here by the characterization of the different conformational and aggregational properties of the three peptides, namely the 26–57 WT peptide, 26–57 K35L, and 26–57 K48L, together with theoretical predictions. The large increase in β-sheet content and aggregation propensity promoted by the K35L mutation is sequence-specific and not due to a nonspecific change in the electrostatic properties of the WT sequence because the K48L change did not have a noticeable impact on these properties (Figs. 3–5). These data support Lys-35 acting as a gatekeeper residue that diminishes the aggregation propensity of the adjacent sequence. In contrast to Lys-48, which is fully exposed, the side chain of Lys-35 is partially shielded in the native tetrameric structure, suggesting that this gatekeeping effect might constitute a safeguard mechanism to reduce aggregation in the case where dissociation occurs and conformational fluctuations might expose the hydrophobic residues Val-28, Ala-29, Val-30, Val-32, and Phe-33, all them buried in the native structure.

Three other positive charges are present in the 26–57 stretch studied here (His-32, Arg-34, and His-56). Whereas H56L substitution did not have any impact on the aggregation propensity of TTR (data not shown), His-32 and Arg-34 are already included in the highly aggregation-prone region of TTR (region 26–34; see Fig. 3, *A* and *B*). Lys-35 flanks this region, reinforcing its role as a gatekeeper residue, and its substitution by leucine extends the aggregation-prone region (Fig. 3*B*).

Organic solvents and detergents are used to simulate a specific cellular environment or to induce a particular protein conformation that triggers the formation of aggregates (56–58). Interestingly, the formation of amyloid fibrils by the only other two TTR peptides characterized to date, corresponding to the A and G strands and comprising TTR residues 10–20 and 105–115, respectively, requires the presence of acetonitrile as a co-solvent (59–61). Here, we showed that co-solvents, such as acetonitrile, TFE, and DMSO, were effective in accelerating WT peptide aggregation (Fig. 2*A*). The ability of SDS to induce and stabilize α-helical structure in peptides is well documented (62), and in agreement, we could force the aggregation of the WT peptide through a nucleation-polymerization reaction by the addition of SDS (Fig. 2, *A* and *B*). The reaction involved the formation of a highly helical intermediate in the nucleation phase (Fig. 2*C*), which did not bind Th-T (Fig. 2*B*). TEM at early time points of the aggregation of the WT peptide in the presence of SDS showed the absence of any aggregated material, which suggests that this α-helical intermediate is probably monomeric (Fig. 2*E*). An α-helical to β-sheet transition on the pathway to amyloid formation has been observed previously at least for the peptide amyloid-β (63). The AGADIR algorithm (64) predicts a low intrinsic α-helical propensity for this segment in the context of the complete TTR sequence (data not shown), suggesting that this conformation is unlikely to be populated under physiological conditions, in agreement with the absence of any detectable conformational transition or aggregation for this TTR region in the absence of additives (Figs. 2 and 4). Interestingly, Triton X-100, a non-ionic surfactant, did not exert any effect on the aggregation of WT peptide (Fig. 2*A*, *buffer 7*), which suggests that charge interactions are necessary for aggregation induction.

The sigmoidal profile of the aggregation kinetics of the K35L peptide at 25 °C (Fig. 6*A*) strongly suggests the existence of a nucleation process by which small, soluble oligomers or nuclei are formed prior to the polymerization reaction (42, 43). The addition of nuclei is generally followed by a strong acceleration of the aggregation reaction because, despite having a certain degree of β-sheet structure, these species still display a significant hydrophobic exposed surface area able to rapidly sequester soluble species to the aggregation pathway (4, 65). These species have been shown to be the major cytotoxic agents in many amyloid diseases (5, 6, 66). Interestingly, although the addition of these preformed oligomers accelerated the aggregation of soluble K35L peptide (Fig. 6*D*), the addition of seeds (sonicated mature fibrils) did not (not shown). This observation has important implications for the mechanism of aggregation of the WT peptide and probably WT-TTR, because it suggests that the main role of the Lys-35 residue is precluding the population of small oligomeric species, probably through the repulsive effect of the two adjacent positive charges (Arg-34 and Lys-35), because once these small assemblies are populated, they become very powerful in accelerating the aggregation reaction. The corollary is that the WT sequence does not aggregate significantly simply because it cannot access the oligomeric competent conformation. In this context, any change/co-solvent that disturbs the gatekeeping action of Lys-35 is expected to have a large impact on aggregation.

SEC (Fig. 6*B*) and CD (Fig. 6*C*) experiments suggested, respectively, that the nuclei formed during aggregation of K35L peptide have a molecular mass of ∼40 kDa, being rich in β-sheet structure. Considering the mass of the peptide (∼3440 Da), we can assume that the nucleus encompasses ∼10–15 units, and, as shown by TEM (Fig. 6, *E* and *F*), it presented the typical morphology previously observed during aggregation of other amyloidogenic proteins described in the literature (44–46).

We confirmed the importance of the hydrophobic character of Leu-35 and its involvement in the K35L peptide fibril core by performing digestion reactions with two proteases, followed by mass spectrometry sequencing (Table 2). As a result of the digestion of both proteases, the residue Leu-35 is incorporated into the protected region of the fibrils. The absence of an additional cleavage after Lys-48 by trypsin might imply either that the core of the fibril expands up to this region or that the enzyme was unable to access it due to the proximity of the fibril core, which would impede docking of this lysine into the trypsin active site.

The results obtained with the 26–57-TTR peptides and discussed so far point to the role of Lys-35 as a gatekeeper residue on the TTR sequence. To confirm this hypothesis, we produced recombinantly and mutated the full-length TTR (K35L-TTR) and performed classical stability and aggregation assays. In agreement with what is expected for a gatekeeper residue, it did not change protein stability (Fig. 7*A*), allowing correct folding and function, but when aggregation was triggered by environment, in this case low pH, it exercised its function, by diminishing propensity for aggregation (Fig. 7*B*).

How does heparin override the gatekeeper? Glycosaminoglycans are naturally abundant in the extracellular matrix and have been shown to be associated with amyloid deposits in a number of amyloidoses, and accumulating evidence suggests that they play an important role in the process of amyloid formation. Heparan sulfate and especially heparin facilitate, *in vitro* and *in vivo*, TTR fibrillogenesis, although the exact mechanism underlying this effect is still under debate (52, 53). Noborn *et al.* (32) dissected the TTR sequence in 16 peptides and analyzed their binding to heparin. Among the peptides tested, only the one encompassing residues 24–35 (PAINVAVHVFRK) was able to efficiently bind heparin (32). Thus, it was suggested that binding to this region probably accounts for accelerated TTR fibrillogenesis in the presence of heparin. This region contains three basic amino acids in close proximity, His-31, Arg-34, and Lys-35, whose positive charges are thought to be the main contributors to heparin binding (67). In particular, the protonation state of His-31 would account for the pH dependence of the TTR-heparin interaction, which occurs at mildly acidic conditions (pH 5.0) but not at neutral pH (pH 7.4). Interestingly enough, the 24–35 TTR sequence stretch overlaps significantly with the WT peptide studied here.

Our results show that presence of heparin allows bypassing of the gatekeeping effect of Lys-35 on WT peptide aggregation (Fig. 8) as well as on full-length WT-TTR aggregation (Fig. 7*B*). In fact, Lys-35 is one of the three basic residues in TTR probably involved in heparin binding, a property that is not shared by Lys-48, because the 41–50 TTR fragment does not bind to heparin (32). Binding of heparin will neutralize the charge of both Arg-34 and Lys-35 and would allow the highly aggregation-prone region adjacent to these residues to express its intrinsic amyloidogenic potential. Heparin binds preferentially to dissociated TTR, which suggests that the heparin-binding domain of TTR is cryptic in the natively folded tetrameric structure (32). In fact, the residues comprising the B β-strand, including His-31, Arg-34, and Lys-35, expose less than 25% of their surface to solvent in the folded state (Swiss- PdbViewer) (68). Thus, the predicted aggregation-prone region and the heparin-binding domain overlap significantly, are protected in the native structure, and would become exposed to solvent only upon intermediate formation. In the absence of heparin, Lys-35 prevents the self-assembly of this protein region both at neutral and at acidic pH because the substitution of this residue by Leu results in fast aggregation at physiological temperature at both pH. The gatekeeping effect exerted by the positively charged side chain of Lys-35 in wild type TTR is further supported by the existence of two amyloidogenic, disease-linked, natural mutations in which the charge of this residue is lost, K35T and K35N (69, 70). In the presence of heparin, the protective effect of Lys-35 is kept at neutral pH, where heparin has a negligible affinity for TTR but is abolished at mildly acidic pH, where it binds this region. Although much more work is needed, these findings would open the possibility of designing new potential therapeutic agents against TTR-related amyloidoses.

## Footnotes

3 The abbreviations used are:

FAP: familial amyloid polyneuropathy
SSA: senile systemic amyloidosis
TEM: transmission electron microscopy
TTR: transthyretin
Th-T: Thioflavin T
PK: proteinase K
SEC: size exclusion chromatography
TFE: trifluoroethanol.
